# Molecular prevalence of *Anaplasma marginale*, *Babesia bigemina,* and *Theileria orientalis* and their associations with Mafriwal cattle’s age groups

**DOI:** 10.5455/javar.2024.k787

**Published:** 2024-06-09

**Authors:** Muhamad Ali Hanapiah Ab. Manap, Nur Sabrina Ahmad Mustaza, Nur Amalina Nasruddin, Wan Ladiana Wan Abdullah, Halimatun Yaakub, Basripuzi Nurul Hayyan

**Affiliations:** 1 Department of Veterinary Paraclinical Studies, Faculty of Veterinary Medicine, Universiti Malaysia Kelantan, Kota Bharu, Kelantan, Malaysia; 2 Pusat Ternakan Haiwan Air Hitam, Kluang, Johor, Malaysia; 3 Department of Animal Science, Faculty of Agriculture, Universiti Putra Malaysia, Serdang, Selangor, Malaysia; 4 UMK Veterinary Diagnostic Centre, Faculty of Veterinary Medicine, Universiti Malaysia Kelantan, Kota Bharu, Kelantan, Malaysia

**Keywords:** *Anaplasma marginale*, *Babesia bigemina*, *Theileria orientalis*, hemoparasite, Mafriwal cattle

## Abstract

**Objective:**

This study was designed to determine the molecular prevalence of hemoparasites and their associations with Mafriwal cattle’s age groups.

**Materials and Methods:**

Blood samples were taken from the coccygeal veins of calves (*n = *92), yearlings (*n = *95), lactating (*n = *90), and dry (*n = *94) cows, which were subjected to microscopic and molecular identification of hemoparasites. The prevalence rate was determined based on the proportion of infected samples in the observed samples. Associations between hemoparasitism and different age groups of Mafriwal cattle were determined by the odds ratio and Fisher’s exact test.

**Results:**

*Babesia bigemina* was the most prevalent hemoparasite in monospecies infection (20.8%), while the co-infection of *Anaplasma marginale* and *B. bigemina* (36.4%) had the highest molecular prevalence. Highly significant associations of hemoparasitism were observed between calves and yearlings (*p < *0.001, Odds ratio = 21.340, 95% CI = 3.200–907.871), lactating (*p < *0.01, Odds ratio = 6.600, 95% CI = 1.808–36.516), and dry (*p < *0.001, Odds ratio = 10.457, 95% CI = 2.363–96.242) cows. Nevertheless, calves and yearlings were 2–4 times more likely to be co-infected with multiple hemoparasite species in comparison to older age groups.

**Conclusion:**

Mafriwal cattle were more susceptible to hemoparasitism with advancing age, but the younger calves were more prone to be co-infected with multiple hemoparasite species.

## Introduction

Hemoparasitism has become one of the most important ruminant diseases all over the world that affects their production, health, and welfare. The disease, which is also categorized as a tick-borne disease, is worsened by the presence of asymptomatic carriers, who play a significant role as the infection source for susceptible ruminants [1]. According to Burrow et al. [2], approximately 80% of the worldwide cattle population is affected by the disease. Economic losses from hemoparasitism in the beef cattle industry are estimated to be US$34.61 per cow in the backgrounding phase and US$7.97 per cow in the finishing phase, respectively [3]. Anemia, reduced weight gain, jaundice, low production, problems in reproduction, and high morbidity and mortality are the common signs of hemoparasitism [4].

Examination of thin blood smears microscopically is a common technique to detect hemoparasite infections, but it is ineffective for species identification. The molecular method, with its high specificity and sensitivity, enables better detection, particularly among asymptomatic and carrier animals with low parasitemia [5]. *Anaplasma marginale, Babesia bovis, Babesia bigemina, Trypanosoma evansi, Theileria orientalis, *and* Candidatus Mycoplasma hemobos* are hemoparasites that have been identified in cattle farms in Malaysia [5,6].

The hemoparasite species are transmitted to cattle by common arthropod vectors from various genera of ticks and flies such as *Dermatocenter *sp., *Hyalomma* sp., *Boophilus* sp., or *Rhipichepalus* sp., as well as the Tabanus family and *Stomoxys* sp. [7,8]. In total, as many as 2803 blood samples have been submitted to the Central Veterinary Laboratory Malaysia for the diagnosis of hemoparasite infections from 2011 to 2015 [8].

On the host’s part, Mafriwal cattle derive their name from the Malaysian Friesian-Sahiwal crossbred that has been developed by the Department of Veterinary Services (DVS), Malaysia, since 1974 for the dual-purpose of meat and milk production under the National Dairy Development Programme [9]. The program envisioned developing a dairy breed with a high milk yield that was adapted to the tropical environment. The selection traits of Mafriwal cattle include 50% for milk production based on solid non-fat, 25% for meat production based on average weight gain, feed conversion ratio, and carcass quality, 15% for utility such as heat tolerance, tick resistance, temperament, and conformation, and 10% for reproduction based on first calving age and calving-conception interval. Mastura et al. [10] also listed resistance to local diseases as one of the desired characteristics of Mafriwal cattle.

Although there was evidence that body weight and milk production of Mafriwal cattle have been significantly affected by *A. marginale, B. bigemina, *and* T. orientalis* [11], the crucial aspect of determining the most susceptible age group within this cattle breed remains conspicuously unexplored, leading to ineffective control approaches that were supposedly implemented to target the susceptible age group. Therefore, this research was performed to determine the molecular prevalence of hemoparasites and their associations with Mafriwal cattle’s age groups. It aimed to reveal whether susceptibility occurs predominantly in a specific age group and whether such susceptibility manifests through monospecies infection or co-infection of multiple hemoparasite species, thus addressing a notable gap in current knowledge.

## Materials and Methods

### Location, duration, and animal ethics approval

This study was conducted from April 2020 to May 2022 at a government commercial dairy farm in Kluang, Johor, which is located in the southern region of Peninsular Malaysia (2°2′1′′N, 103°19′10′′E). Approval by the Institutional Animal Care and Use Committee (IACUC) of the Faculty of Veterinary Medicine, Universiti Malaysia Kelantan (UMK/FPV/ACUE/RES/2/2020) has been obtained before the commencement of the study.

### Management of cattle

The Mafriwal cattle groups consisted of calves less than 1-year-old, yearlings, lactating cows, and dry cows. Newborn calves were given colostrum at 10% of their body weight twice a day for seven days before being separated into individual pens. Then, the calves were bottle-fed with raw, fresh milk at 10% of their body weight. The calves were introduced to cut grasses and calf starter pellets at 2 months old. The concentrates were added to the diet until 100 days or when the calves reached a body weight of 90 kg for weaning. The calves were managed intensively until they reached 1-year-old (yearlings) before being released into the signal (*Brachiaria decumbens*) and guinea (*Panicum maximum*) grass paddock with a rotational grazing system. The yearlings were provided with concentrates and supplements such as palm kernel cakes and molasses once a day as additional nutrition. Later, the 2-year-old heifers were mixed with selected bulls on the pasture and subsequently proceeded into gestation, lactation, and dry cow groups. However, the gestation group was excluded from this study to avoid stress during sampling.

### Blood sampling and microscopic screening for hemoparasites

A total of 371 Mafriwal cattle of different age groups were sampled throughout the study. The sampling was conducted once per year from 2020 to 2022 (Table 1). Blood from each cattle was sampled from the coccygeal vein into ethylenediaminetetraacetic acid (EDTA) tubes. Fresh blood samples were screened microscopically in the field laboratory for the presence of hemoparasites in the first year of sampling. Thin blood smears were prepared on the same day of sample collection following a standard procedure by the DVS Malaysia [12,13]. Diff-Quik solutions were used for staining according to the manufacturer’s protocol (Labchem Sdn. Bhd., Malaysia). Each slide was screened for the presence of hemoparasites under a light microscope at 40x and 100x magnification (Olympus CX21, Japan) based on the morphology described by Atif [14], Sivakumar et al. [15], Brahma et al. [16], and Chandrawathani et al. [13]. The remaining blood samples were transported to the Faculty of Veterinary Medicine, Universiti Malaysia Kelantan, and kept at −20°C for further identification of hemoparasite species by molecular method.

**Table 1.** Number of sampled Mafriwal cattle based on age groups between 2020 and 2022.

Year	Cattle groups				Total
	<1-year-old calves	Yearlings	Lactating cows	Dry cows	
2020	32	34	29	34	129
2021	30	31	31	30	122
2022	30	30	30	30	120
Total	92	95	90	94	371

### Molecular identification of hemoparasites by polymerase chain reaction

Deoxyribonucleic acid (DNA) extraction of hemoparasites was conducted using Geneaid Gsync™ DNA extraction kits based on the protocol from the manufacturer (Geneiad Biotech Ltd., New Taipei City, Taiwan). The extracted DNA was stored at −20°C and subjected to polymerase chain reaction (PCR) amplification by MyCycler™ thermocycler (Bio-Rad, USA) according to the species-specific primer sets and thermocyclic profiles of bovine hemoparasites in Peninsular Malaysia [6], namely *A. marginale, B. bigemina *and* T. orientalis,* that have been used for the detection of hemoparasite species in Mafriwal cattle [11]. The PCR products of the reaction of the DNA template (5.0 μl), each primer (1.0 μl), nuclease-free water (1.5 μl), and 1x GoTaq Green Master Mix (12.5 μl) (Promega Madison, USA) were electrophoresed at 100 v for 40 min [11]. Midori Green Dye (Nippon Genetics, Europe) was used to stain the Tris-acetate-EDTA buffer, and the result was visualized using the GelDoc™ EZ Imager. The DNA fragments of each hemoparasite species were purified using the Gel/PCR DNA Fragment Extraction Kit (Geneaid Biotech Ltd., New Taipei City, Taiwan). The PCR products were submitted for Sanger sequencing (Apical Scientific Sdn. Bhd., Malaysia) before being subjected to nucleotide identification by the basic local alignment search tool in the GenBank of the National Center for Biotechnology Information (NCBI).

**Figure 1.**
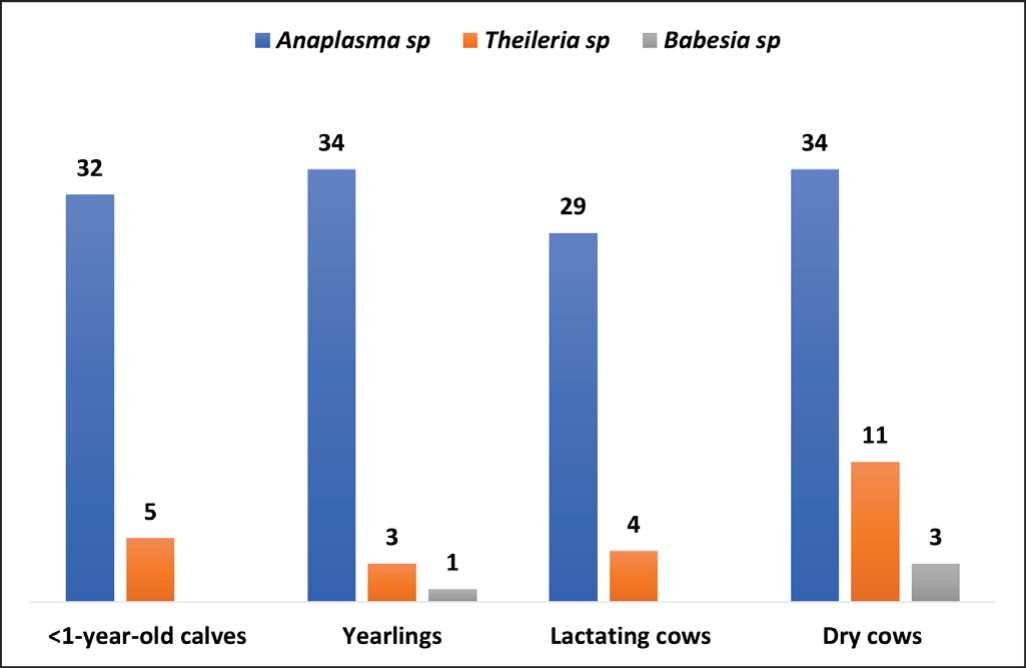
Numbers of different age groups of Mafriwal cattle screened microscopically for different genera of hemoparasites in 2020 (*n = *129).

### Statistical analysis

The molecular identification of monospecies and co-species infections of hemoparasites was determined based on age groups (calves less than 1-year-old, yearlings, lactating cows, dry cows) and years of sample collection (2022, 2021, 2022). The prevalence rate was calculated based on the proportion of infected blood samples from the observed blood samples during the molecular identification of hemoparasite species. Odds ratio and Fisher’s exact test were conducted to determine the associations between hemoparasite infection and different age groups of Mafriwal cattle at an alpha value of *p < *0.05. The statistical tests were also applied to determine if the susceptibility of Mafriwal cattle to hemoparasite infection was associated with a particular species or the co-infection of multiple hemoparasite species. The R software version 4.2.2 was used to conduct the statistical analyses.

## Results

### Microscopic screening of hemoparasite genera on thin blood smears

All of the observed thin blood smears from different age groups of Mafriwal cattle in 2020 were detected with *Anaplasma* sp. (*n = *129, 100%) (Fig. 1). *Theileria *sp. were also detected in the thin blood smears of all age groups, with the highest detection rate in dry cows (*n = *11, 32.4%) and the lowest detection rate in yearlings (*n = *3, 8.9%). However, *Babesia* sp. was not detected microscopically in lactating cows and calves less than 1 year old or younger. This genus was only detected in three dry cows (8.8%) and a yearling (2.9%).

### Molecular prevalence of hemoparasite infections from 2020 to 2022

**Figure 2.**
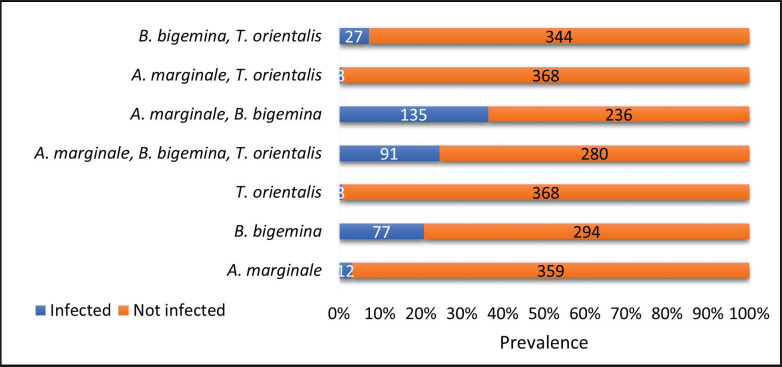
Molecular prevalence of hemoparasite species with the numbers of infected and non-infected Mafriwal cattle from 2020 to 2022 (*n = *371).

Throughout three years of study on a total of 371 Mafriwal cattle, *B. bigemina* was detected as the most prevalent hemoparasite among monospecies infections (*n = *77, 20.8%) in comparison to *A. marginale* (*n = *12, 3.2%) and *T. orientalis* (*n = *3, 0.8%) (Fig. 2). The highest molecular prevalence was estimated for the mixed infection of *A. marginale *and *B. bigemina* (*n = *135, 36.4%), followed by a co-infection of all hemoparasite species identified in this study, namely *A. marginale, B. bigemina, *and* T. orientalis* (*n = *91, 25.6%). The lowest molecular prevalence was detected in monospecies infections of *T. orientalis *and co-infections of *A. marginale *and* T. orientalis* (*n = *3, 0.8%).

**Figure 3.**
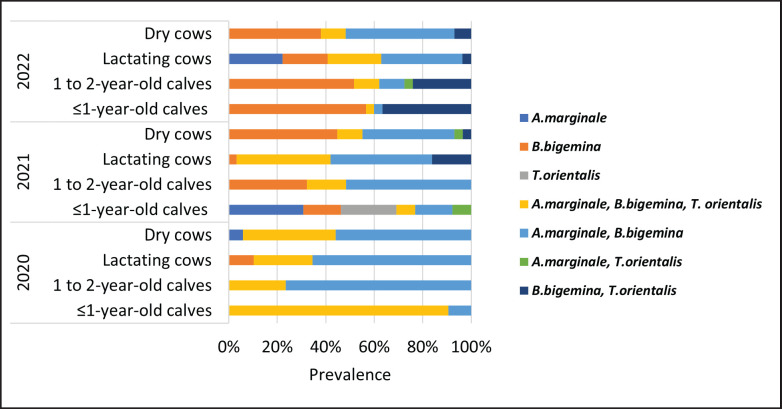
Molecular prevalence of hemoparasite species based on year of sampling and age groups of Mafriwal cattle (*n = *371).

Based on the year of sampling (2020–2022) and age groups of Mafriwal cattle (calves of less than 1-year-old, yearlings, lactating cows, and dry cows) (Fig. 3), the group of calves of less than 1-year-old in 2020 showed the highest molecular prevalence of co-infection of all identified hemoparasites (*n = *29, 85.3%). This was followed by the groups that were co-infected with *A. marginale *and* B. bigemina, *namely the yearlings (*n = *26, 76.5%), lactating cows (*n = *19, 65.5%), and dry cows (*n = *19, 55.9%) in the same year. The molecular prevalence of *A. marginale, B. bigemina*, and* T. orientalis* co-infection in dry cows was also noticeable in 2020 (*n = *13, 38.2%).

Co-infection of *B. bigemina* and *A. marginale* was considered predominating hemoparasite infections among Mafriwal cattle in 2021, with the highest molecular prevalence in calves of 1 to 2-year-old (*n = *16, 51.6%), followed by lactating cows (*n = *13, 41.9%), and dry cows (*n = *11, 36.7%) (Fig. 3). Co-infection of all identified hemoparasites was also prevalent in lactating cows (*n = *12, 38.7%). Additionally, *B. bigemina* was the most prevalent monospecies in calves of 1 to 2-year-old (*n = *10, 32.3%) and dry cows (*n = *13, 43.3%) in 2021.

**Table 2.** Pairwise comparison between different age groups of Mafriwal cattle on hemoparasite infection from 2020 to 2022.

Comparison groups	n*	95% CI^†^	*p-*value	Odds ratio
Dry cows Lactating cows	92/94 87/90	0.177–19.362	0.677	1.582
Dry cows Yearlings	92/94 94/95	0.008–9.586	0.621	0.491
Dry cows ≤1-year-old calves	92/94 74/92	2.363–96.242	1.685e-04	10.457
Lactating cows Yearlings	87/90 94/95	0.006–3.947	0.358	0.310
Lactating cows ≤1-year-old calves	87/90 74/92	1.808–36.516	1.453e-03	6.60
Yearlings ≤1-year-old calves	94/95 74/92	3.200–907.871	2.637e-05	21.340

*n* = number of infected cattle over non-infected cattle, CI = confidence interval.

**Table 3.** Pairwise comparison between different age groups of Mafriwal cattle on monospecies infection and co-infection of multiple hemoparasite species from 2020 to 2022.

Hemoparasite	Age groups	n*	95% CI^†^	*p*-value	Odds ratio
*A. marginale, B. bigemina,* *T. orientalis*	<1-year-old calves Yearlings	31/92 16/95	1.199–5.364	0.011	2.497
	<1-year-old calves Lactating cows	31/92 25/90	0.670–2.617	0.424	1.319
	<1-year-old calves Dry cows	31/92 19/94	0.984–4.140	0.047	1.999
*A. marginale* *B. bigemina*	Yearlings <1-year-old calves	45/95 6/92	4.943–39.096	1.013^e-10^	12.722
	Yearlings Lactating cows	45/95 41/90	0.579–1.997	0.833	1.146
	Yearlings Dry cows	45/95 74/94	0.579–1.968	0.884	1.067
*B. bigemina* *T. orientalis*	<1-year-old calves Yearlings	11/92 7/95	0.571–5.444	0.329	1.702
	<1-year-old calves Lactating cows	11/92 6/90	0.608–6.546	0.309	0.309
	<1-year-old calves Dry cows	11/92 3/94	1.032–23.628	0.027	4.091
*A. marginale*	<1-year-old calves Yearlings	4/92 0/90	-	-	INF
	<1-year-old calves Lactating cows	4/92 6/95	0.128–2.799	0.638	0.534
	<1-year-old calves Dry cows	4/12 2/12	0.290–23.569	0.442	2.083
*B. bigemina*	Yearlings <1-year-old calves	25/95 19/92	0.659–2.886	0.392	1.370
	Yearlings Lactating cows	25/95 9/90	1.334–8.323	0.004	3.195
	Yearlings Dry cows	25/95 24/94	0.516–2.106	1.000	1.041

*n* = number of infected cattle over non-infected cattle, CI = confidence interval.

Figure 3 also shows that *B. bigemina* was identified as the most predominant hemoparasite species in 2021 among calves of 1-year-old or calves less than 1-year-old, calves between 1 and 2-year-old, and dry cows, with a molecular prevalence of 56.7% (*n = *17), 50% (*n = *15), and 36.7% (*n = *11), respectively. Co-infections of two hemoparasite species were also prevalent in dry cows infected with *A. marginale *and* B. bigemina* (*n = *13, 43.3%) as well as in calves less than 1-year-old or less that were infected with *B. bigemina *and* T. orientalis* (*n = *11, 36.7%).

### Comparative sequence analysis of hemoparasite species

The representative nucleotide sequences from each detected hemoparasite displayed 98–100% similarities with the sequences published in the NCBI GenBank database. Five major surface protein-4 nucleotide sequences (Accession No. OP437580–OP437584) have 100% similarities with *A. marginale* isolates from cattle in Thailand (Accession No. MK140740.1) and Cuba (Accession No. MK809387.1). Only one apical membrane antigen-1 nucleotide isolate (Accession No. OP437585) was found with 98.36% similarities to the *B. bigemina* isolate from cattle in Sri Lanka (Accession No. LC438495.1). In contrast, four others (Accession Nos. OP437586–OP437589) have 100% similarities with the same isolate. Three major piroplasm surface protein nucleotide sequences (Accession no: OP965415-OP965417) showed 100%, 99.45%, and 99.51% identity with *T. orientalis* isolates from cattle in Sri Lanka, Thailand, and India (Accession no. LC438487.1, KU886279.1, and MK874825.1), respectively.

### Association of Mafriwal cattle’s age groups with hemoparasite infection

Highly significant associations were observed between the youngest age group of Mafriwal cattle (<1-year-old calves) and the older age groups, namely the yearlings (*p < *0.001, Odds ratio = 21.340, 95% CI = 3.200–907.871), lactating cows (*p < *0.01, Odds ratio = 6.600, 95% CI = 1.808–36.516) and dry cows (*p < *0.001, Odds ratio = 10.457, 95% CI = 2.363–96.242) that were molecularly detected with at least a hemoparasite species from 2020 to 2022 (Table 2). No significant associations were observed between yearlings, lactating cows, and dry cows.

Table 3 shows that the youngest age group was 2.5 times more susceptible to the co-infection of all identified hemoparasites than the yearlings (*p < *0.05, Odds ratio = 2.497, 95% CI = 1.199–5.364). The youngest age group of Mafriwal cattle was also susceptible to the co-infection of these hemoparasites at 2 times higher than the dry cows (*p < *0.05, Odds ratio = 1.999, 95% CI = 0.984–4.140). In comparison to the dry cows, the youngest age group was four times more likely to be co-infected with *T. orientalis* and *B. bigemina *(*p < *0.05, Odds ratio = 4.091, 95% CI = 1.032–23.628). On the other hand, the yearlings were 12.7 times more vulnerable to the co-infection with *A. marginale *and* B. bigemina* than the calves of less than 1-year-old (*p < *0.001, Odds ratio = 12.722, 95% CI = 4.943–39.096). In addition, the yearlings were 3.2 times more prone to *B. bigemina* monospecies infection than the lactating cows (*p < *0.01, Odds ratio = 3.195, 95% CI = 1.334–8.323). Nonetheless, it is important to note that the odds ratio of *A. marginale* infection between calves less than 1-year-old and yearlings reached an infinite value, as none of the yearlings were detected to have this hemoparasite in monospecies infection. As monospecies infection of *T. orientalis* only occurred among calves less than 1-year-old, pairwise comparisons with the other age groups were not possible in this study.

## Discussion

This research revealed the occurrence of anaplasmosis, babesiosis, and theileriosis among Mafriwal cattle in a government commercial dairy farm within the three years of study. Since five decades ago, the Mafriwal cattle have been managed according to their age groups. Hence, this study also explored the associations between the age groups of Mafriwal cattle and hemoparasitism caused by either monospecies infection or co-infection of multiple species, namely *A. marginale, T. orientalis, *and* B. bigemina.*

The study discovered distinct patterns of different hemoparasite species infections among Mafriwal cattle, shedding light on the prevalence and distribution of these hemoparasites. A notable finding was the microscopic detection of *A. marginale* in all blood samples in 2020 (Fig. 1), suggesting active infections in the Mafriwal cattle population. This finding was consistent with Ola-Fadunsin et al. [6], where *A. marginale* was highlighted as the most prevalent bovine hemoparasite in Peninsular Malaysia. The *A. marginale* is known as the most infectious species among the Anaplasmataceae family which leads to high morbidity and mortality among livestock, accompanied by severe symptoms such as anemia, lethargy, and inappetence [17].

Further molecular identification discovered that *B. bigemina* predominated monospecies infection, followed by *A. marginale* and *T. orientalis* (Fig. 2), which is supported by the previous study [11]. This finding implies that the cattle were suffering from chronic but low-intensity babesiosis, where the DNA of *B. bigemina* persisted in the infected animals, thus, they could be identified molecularly but undetected microscopically in most samples.

In comparison to monospecies infection, the active infections of *A. marginale* contributed more to co-species infection with *T. orientalis and/or B. bigemina*. Hence, the co-species infection involving *A. marginale* can be divided into three groups: *A. marginale *and* T. orientalis; A. marginale *and *B. bigemina; *and *A. marginale*, *T. orientalis, *and* B. bigemina.* As expected, the co-infection of *A. marginale* and *B. bigemina* predominated the infection, where 135 out of 371 Mafriwal cattle were infected during the study, followed by the co-infection of all identified hemoparasites (Fig. 2).

These findings revealed that *A. marginale* and *B. bigemina* were the most important hemoparasites among Mafriwal cattle. The findings also suggest that co-infection of multiple hemoparasite species was more common in natural infections compared to monospecies infections. This finding is important because the consequences of natural co-infection between two or more parasite species on the health and productivity of livestock will provide a better understanding of host-parasite interaction [18].

There were different patterns of infection based on the age groups of Mafriwal cattle and hemoparasite species in each year of sampling (Fig. 3). Monospecies infections of *T. orientalis* and co-infections of two hemoparasite species involving *T. orientalis *were infrequent, regardless of age groups. These findings were inconsistent with Rohaya et al. [8], in which theileriosis was the most common hemoparasitism diagnosed in cattle between 2011 and 2015 in the central region states of Peninsular Malaysia. This could be due to theileriosis outbreaks that may occur in the central region, which is located farther away from the location of the present study in the south region. Furthermore, the Mafriwal cattle were localized in a dedicated farming area with fenced pastures where the movements of animals and farm personnel were strictly controlled. Therefore, exposure to the vectors of hemoparasites was limited to the cattle already present on the farm with a lower burden of theileriosis.

The age-based analysis discovered intriguing insights, with Mafriwal cattle of more than 1 year old consisting of yearlings, lactating cows, and dry cows displaying higher susceptibility to hemoparasites compared to their younger counterparts. This aligns with findings from various global studies indicating increased susceptibility among adult cattle to hemoparasite infections [17,19,20,21].

Early exposure to hemoparasite infections will contribute to the development of immunity among calves. Juvenile cattle less than 9-month-old have an innate resistance to *Babesia* spp. infections, infrequently show clinical signs and obtain natural immunity during recovery [22]. It is important to note that the calves that were managed intensively on this farm have less exposure to the vectors of hemoparasites in comparison to the older age groups that were released to the pasture. This factor may lead to the development of susceptible animals with advancing age, thus contributing to the higher numbers of infected cattle in the aged groups. Interestingly, lactating and dry cows exhibited lower rates of co-infections compared to the younger age groups, suggesting potential immunity maintenance through continuous vector exposure (Table 3). However, the roles of vectors, such as ticks and blood-sucking flies, were not investigated in this study.

Control strategies should encompass both therapeutic interventions and vector management approaches. While vector eradication is known to be difficult in tropics and subtropical regions due to suitable conditions for multiplication [23], short-duration rotational grazing [24] coupled with routine acaricide applications [25] emerge as cost-effective control measures to reduce population and transmission of the vectors. Additionally, restricting contact with wildlife reservoirs through proper fencing can mitigate transmission risks, although the movements of animals and farm personnel are strictly controlled on this farm.

One potential weakness of this study is the lack of investigation into the specific vectors responsible for transmitting hemoparasites to Mafriwal cattle. Understanding the role of vectors is crucial for developing effective control strategies. Future research that includes vector surveillance and identification could strengthen the study’s findings and provide a more comprehensive understanding of hemoparasite transmission in Mafriwal cattle populations. Besides, the findings may not be fully representative of broader trends in hemoparasite prevalence and distribution due to the limited scope of the geographical area studied. Variations in environmental factors, management practices, and genetic factors across different regions could influence the dynamics of hemoparasite infections in cattle populations. Therefore, expanding the study to encompass multiple geographic locations or farms would enhance the findings.

## Conclusion

In conclusion, anaplasmosis, babesiosis, and theileriosis were prevalent among Mafriwal cattle of different age groups on this farm. Although cattle older than 1-year old were more susceptible to being infected with hemoparasites than the younger calves, the latter were more prone to be co-infected with multiple hemoparasite species than their older counterparts. Therefore, effective strategies should be implemented to control hemoparasite infection among cattle in all age groups.

## List of Abbreviations

µl, microliter; *A. marginale, Anaplasma marginale*; *B. bigemina, Babesia bigemina*; CI, confidence interval; DNA, deoxyribonucleic acid; DVS, Department of Veterinary Service; EDTA, ethylenediaminetetraacetic acid; n, number of animals; NCBI, National Center for Biotechnology Information; PCR, polymerase chain reaction; sp., Species; *T. orientalis*, *Theileria orientalis*.
